# The attitudes of brain cancer patients and their caregivers towards death and dying: a qualitative study

**DOI:** 10.1186/1472-684X-6-7

**Published:** 2007-11-08

**Authors:** Nir Lipsman, Abby Skanda, Jonathan Kimmelman, Mark Bernstein

**Affiliations:** 1 Division of Neurosurgery, Toronto Western Hospital, 399 Bathurst Street, 4W451, Toronto, Ontario, M5T 2S8, Canada; 2 Medical Bioethics Unit, McGill University, 3647 Peel Street, Montreal, Quebec, H3A 1X1, Canada

## Abstract

**Background:**

Much money and energy has been spent on the study of the molecular biology of malignant brain tumours. However, little attention has been paid to the wishes of patients afflicted with these incurable tumours, and how this might influence treatment considerations.

**Methods:**

We interviewed 29 individuals – 7 patients dying of a malignant brain tumor and 22 loved ones. One-on-one interviews were conducted according to a pre-designed interview guide. A combination of open-ended questions, as well as clinical scenarios was presented to participants in order to understand what is meaningful and valuable to them when determining treatment options and management approaches. The results were analyzed, coded, and interpreted using qualitative analytic techniques in order to arrive at several common overarching themes.

**Results:**

Seven major themes were identified. In general, respondents were united in viewing brain cancer as unique amongst malignancies, due in large part to the premium placed on mental competence and cognitive functioning. Importantly, participants found their experiences, however difficult, led to the discovery of inner strength and resilience. Responses were usually framed within an interpersonal context, and participants were generally grateful for the opportunity to speak about their experiences. Attitudes towards religion, spirituality, and euthanasia were also probed.

**Conclusion:**

Several important themes underlie the experiences of brain cancer patients and their caregivers. It is important to consider these when managing these patients and to respect not only their autonomy but also the complex interpersonal toll that a malignant diagnosis can have.

## Background

The issue of the psychological state of the dying has received significant attention in the medical literature [1-9]. Most research has focused on questions surrounding physician assisted suicide, and recently, the increasing awareness of spiritual and existential factors that can contribute to psychological, and physical well-being at the end of life [1,2,10-15]. Although questionnaires and surveys assessing these factors have been validated in various palliative populations [3,16,17], they often fail to capture the subjective experience of dying in these patients. For this reason, patient interviews might provide the best opportunity to explore these sensitive issues in a way that respects the diversity of the illness experience [18-21]. Such a qualitative analytical approach can be used to understand and capture the dynamic state of mind of the dying patient. The purpose of the present investigation was to explore what is meaningful to individuals dying of terminal brain cancer. Questions surrounding the costs and benefits of various treatment options were discussed in one-on-one interviews in order to gain insight into the factors considered important in the lives of these patients.

The consequences of a cancer diagnosis are far-reaching and complex, affecting not only the patient but his/her network of caregivers as well. Several studies have demonstrated the toll that a cancer diagnosis has on families of patients, and the importance of communication both within families and between health care providers and caregivers [22,23]. Treatment decisions are frequently group decisions, made with the underlying assumption that treatment for one may have implications for many. The value of the caregiver's perspective therefore becomes that much more important, and to accurately gauge attitudes towards treatment and dying, these need to be taken into account. Some studies in the literature have begun to consider caregiver attitudes and these have added significant weight to interpretations of the decision making process in terminally ill patients [19,24,25].

Among the malignant diseases, brain cancer is unique in that the organ affected is traditionally viewed as the seat of an individual's literal sense of identity. Philosophical enquiries regarding the manifestation of behaviour and the cognizance of one's existence all involve the brain, an organ around which many more questions exist than answers. As a result, studying these patients' responses and experiences with an illness that threatens their existence is conceptually and practically appropriate. Although few studies have investigated brain cancer populations specifically, some studies have explored coping and the meaning of illness in advanced cancer patients [1,2,4,20]. For example, in some mixed palliative populations studied, hopelessness and desire for death have been linked to physical distress in addition to psychiatric depression [3,8]. Measures have also been devised assessing and measuring meaning and the will to live [15], in addition to the desire to die [7,16,17,26] in these patients, however, investigators rarely, if ever, discuss cancer type as an independent variable. Such studies attempt to gauge attitudes towards death, but do not necessarily examine what these patients value in life.

Cancer of the brain, though relatively rare, is most frequently fatal [27]. The devastating consequences of the disease for patients, families, and caregivers necessitate further exploration in the domain of subjective illness experience and therapeutic decision making [28]. It is therefore important that both investigators and clinicians acquire a better understanding of what is meaningful and valuable to individuals living with terminal brain cancer. Such an understanding, guided by an analysis of the patient's and caregiver's concerns, wishes and beliefs, would contribute significantly to current models of therapeutic management and developing relevant goals in clinical trials for these patients. The objective of our study, therefore, is to examine the illness experience of caregivers and patients living with brain cancer, and to see how those experiences colour attitudes towards treatment. These issues have yet to be explored in this patient population and this study can potentially aid in their comprehensive management.

## Methods

### Design

The present study was a qualitative case study utilizing face-to-face one-on-one interviews with terminally ill patients and/or their caregivers.

### Setting and participants

Participants in the study were patients with known terminal brain cancer. Where patients could not be interviewed, caregivers and family members were substituted. Patients and families were selected by a senior staff neurosurgeon (MB) and informed consent was obtained from all participants. The study rationale and objectives were comprehensively discussed. Patients with the diagnosis of glioblastoma or metastatic brain cancer were selected and the following exclusion criteria were used: 1) those who were not cognitively intact; 2) those who could not adequately communicate in English; and 3) those felt to be too psychologically fragile to participate. The same inclusion criteria were used to screen caregivers and family members participating in the study.

### Sample size

Twenty-nine (29) participants were sought for interviews, and this included twenty-two caregivers and seven patients. The sample size is similar to previous qualitative studies done with surgical patients [29]. Further, it was felt that the sample size was adequate to reach a sufficient level of "saturation", a term describing a point beyond which no new concepts will arise as a result of further interviews [30]. Also, given the effect of the disease on the caregiver perspective, it was not seen as deleterious to the rigor of the study that the majority of interviews were conducted with close family members.

### Data collection

**Figure 1:**
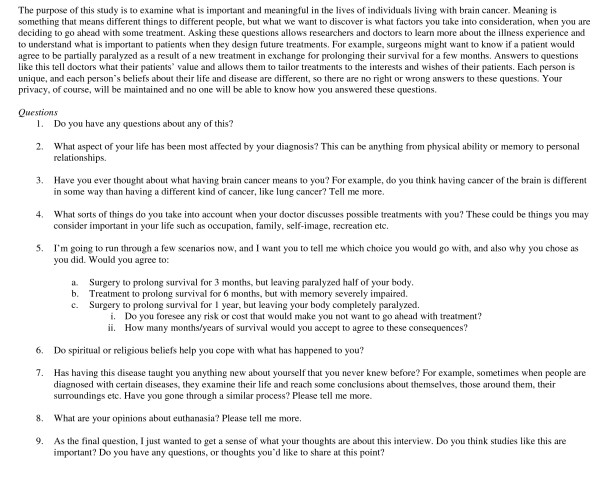
Interview Guide.

This study took place in a tertiary care, teaching hospital, functioning within a socialized medical system. Interviews, which ranged in length between 45 minutes and one hour, were conducted over a period of 12 months. Various aspects of the illness experience were explored, including the role of faith and spirituality, attitudes towards euthanasia and the value of such discussions. Interviews were based on an interview guide (Figure 1), but enough latitude was left to allow the interviewer a chance to explore potentially insightful avenues. The guide was designed by the research team, and continually refined, according to the study's overall objectives. Scenarios contained within the interview guide were strictly hypothetical, although addressed issues that neurosurgical patients are routinely confronted with. Our goal was to examine the treatment decision-making and risk evaluation process in this specific population. A trained research assistant conducted all the interviews. All interviews were audiotaped and transcribed, and demographic information about each patient and caregiver was collected.

### Data analysis

Data were analyzed using modified thematic analysis techniques which included open and axial coding phases [30]. In open coding, data was analyzed and broken down into common groupings based on shared ideas, whereas with axial coding, data was classified according to overarching themes. A computer program was not used for the analysis. Four persons independently participated in the analysis of interview transcripts and in the development of the coding framework. Researchers fractured interview responses and results into ideas and coalesced these into overarching, general themes. Once data was analyzed and interpreted, it was clear that our sample size was sufficient since saturation appeared to have been reached, and no new themes emerged from further analysis.

### Research ethics

Participation in this study was voluntary and all information gathered, including demographics and patient participation, was kept confidential. The study was initially introduced by the treating neurosurgeon, and the details of the study were discussed at a separate time by different members of the research team. Informed consent was obtained at that time by the research assistant who also conducted the interviews. Interview audiotapes and transcripts were anonymously labeled and secured. The Research Ethics Board of the University Health Network approved the study.

## Results

### Participant information

Patient and caregiver demographic data are summarized in Table 1.

**Table 1:** Participant Demographics (N = 29)

*Age*		45.5 (mean)
*Sex*	Male	8 (27.6)*
	Female	21 (72.4)

*Marital status*	Single	4 (13.8)
	Married	20 (70)
	Divorced	3 (10.3)
	Widowed	2 (6.9)

*Education*	Secondary	4 (13.8)
	University/College	21 (72.4)
	Post-graduate	4 (13.8)

*Religion*	None	2 (6.9)
	Hindu	1 (3.4)
	Roman Catholic	12 (41.4)
	Anglican	3 (10.3)
	Christian	2(6.9)
	Jewish	2(6.9)
	Sikh	1 (3.4)
	Muslim	1 (3.4)
	Protestant	3 (10.3)
	Lutheran	1 (3.4)
	Non-religious but spiritual	1 (3.4)

*Numbers in brackets represent percentages of total.

### Thematic analysis

Several themes emerged from the interviews. Caregivers were frequently more forthcoming and reflective than patients, and presumably viewed the interview session as an opportunity to explore these personal issues. The major themes identified shed much light on the experiences of caregivers and patients, and their attitudes are quite informative in the context of a terminal illness affecting the brain. The following is a description of these themes illustrated with verbatim quotes.

### Brain cancer is unique

There appeared to be a relative consensus amongst the study participants that even when compared with the other malignant diseases, brain cancer is unique. Usually, the focus was on the brain as a control center, and the seat of the literal sense of identity, as well as memory and concentration.

"I would probably be concerned about losing the things that make me who I am."

"...the brain is the big center of everything...."

In addition to finding brain cancer unique, this was usually, but not always interpreted in a negative fashion. For example, the rapidity of the intellectual and physical decline, the lack of any effective and long-lasting treatments, and the grimness of the diagnosis were all cited as distinguishing negative features of the disease. Some individuals, however, mentioned cognitive decline, memory loss and the relative lack of pain as *positive *features of the disease that, in effect, spared their loved ones of the awareness of suffering.

"In some ways it's better... [the patient] did not realize what was really happening to him."

These examples speak to the tremendous variation in patient and caregiver experiences. A devastating symptom for one patient, may indeed be a redeeming feature for another patient, or at least provide some emotional or psychological comfort.

Most participants implicitly understood that diseases of the brain are in fundamental ways different than diseases of other organ systems. Frequently, the sentiment was echoed that each brain cancer is unique, whereas other cancers are largely alike.

"If you have lung cancer...your symptoms are usually pretty similar to the other guy that has lung cancer."

"You can remove a breast and you can still function, or you can still work with one lung, and this just seems like its all or nothing."

Perhaps the most powerful, and moving, indictment of the disease came from one participant, who when asked if they could articulate how brain cancer may be different from other cancers, responded:

"It's not just a diagnosis, it's almost like it's a sentence on all the other aspects of his life."

Although several subjects provided the proviso that malignant diseases are terrifying despite the organ involved, there nevertheless remained an overall understanding amongst nearly all participants, that brain cancer is somehow unique and in a category all unto its own.

### Lessons are derived from experiences

Most participants were able to describe lessons that they have learned as a result of dealing and coping with a terminal illness. Some caregivers framed their experiences as challenges or opportunities to learn and grow.

" [The illness experience] Taught me how to face adversity and not feel sorry for myself, and just fight and be strong, and it just gave me a good example."

" [I've learned] I can cope with this, I can deal with this, and in a way that's been empowering for me...I've had to rise to the challenge."

The rapidity of the illness resulted in some patients and caregivers having to significantly adjust their lives and their schedules over a short period of time. Obligations that didn't exist previously, now took precedence, and participants found that they learned to prioritize, and focus on family, and home life.

"I think my life has slowed down back to a point where I can just go through life and enjoy every moment."

The idea of strength emerged as particularly important, as several patients reported surprise at the amount of emotional and psychological fortitude they possessed when dealing with difficult situations.

"I always knew I had strength and that people can lean on me, and it just emphasized that even more."

Again, individual experiences are varied, and not all patients derived lessons from their illnesses. Whether due to pre-existing coping mechanisms or individual personalities, some caregivers and patients approached the disease in unique ways. One caregiver, referring to how he and his wife make decisions regarding treatment, commented:

"We tend to make decisions [when] we have as much information as we can...whether that's the purchase of a TV or dealing with brain cancer."

### Quality of life is very important

One of the most consistent themes that emerged during the interviews was the notion that quality of life, the make-up of which varied between patients, was more important than prolongation of life.

"Quality is more important than quantity."

During the interviews, the authors attempted to gauge attitudes towards quality of life through various scenarios. These included selecting between surgery that would prolong life by 3 months but would result in hemiparesis, or surgery for survival of 12 months but that would result in quadraparesis. Patients overwhelmingly selected the 3 month option, citing the importance of quality of life, both for the patient and caregiver.

"I would choose normal function for less time, without any question."

An additional theme identified, which will be discussed in more detail below, dealt with the premium that patients placed on mental competence. Quality of life was frequently framed in terms of whether an individual patient would be a 'vegetable', or be able to think or maintain awareness. In most instances, participants made it clear that such an existence would be inconsistent with a high quality of life, and elected to hypothetically and in some cases, literally, refrain from obtaining additional treatment.

"He wouldn't want to be around for the sake of just being around."

"If there's no quality of life, then neither one of us wants to be alive."

### Spirituality is often helpful

A majority of caregivers interviewed expressed that spiritual, but not necessarily religious, beliefs were able to provide them with comfort and additional strength to cope with their illness.

"I'm not a practicing Catholic...I think it's more a spiritual sense."

In contrast to caregivers, few patients were able to articulate how religion plays a part in their coping, although some referred to the feelings of connectedness between individuals, and the overall sense of the role that even illness has in a larger, potentially divine, plan.

"We believe we're all connected and that there's, a reason for this. This is part of life and everybody has something that they deal with from time to time and this just happens to be ours."

Whether representing a means to adapt to a difficult situation, or a continued pattern of previous observance, it did not appear that religion or spirituality affected treatment choices or how aggressive patients or caregivers were with their illness. In most respects, spirituality was seen more as a means towards understanding, rather than guidance.

"The soul never dies. The body is just like the jacket you are putting on."

No matter what their roles, or the participants' particular willingness to discuss them, spiritual and/or religious beliefs were common in the majority of individuals interviewed, and were in large part seen as a positive part of their experience.

### Mental competence is paramount

Although the interviews made clear that participants placed a premium on quality, as opposed to quantity, of life, the definition of quality can and does vary in the larger population. We discovered that the patients and caregivers interviewed often felt it necessary, particularly when faced with a terminal disease of the brain, to emphasize the importance of mental functioning, orientation and cognition in their definitions of quality of life. The prospect of memory loss and intellectual decline, in most cases, proved to be the most common reason that participants cited to shorten life by declining additional treatment.

"When you don't have the cognitive ability...I think that's where she draws the line."

"The fact that his mental functioning is intact is really...the most importance piece."

This emphasis on mental capacity, again, drew attention to the unique experiences of brain cancer patients, even among the other aggressively malignant diseases. Patients and families understood that a disease affecting the brain, and in essence, the mind, was qualitatively different than diseases of other organs.

"It's another thing not being able to remember who you are or who I am. You know, that to me is different."

"He always said he would prefer to lose a limb than to have his mind go."

An additional, and valuable, perspective was gained on caregiver priorities as well, with disabilities of the body seen as far easier to manage than disabilities of the mind. Caregivers consistently commented that to lose their loved one's identity, memory and awareness would be tantamount to their dying, and that the loss of independence and vitality in a previously very high functioning individual would be psychologically and emotionally traumatic.

"As long as his mind is there...we could go on forever."

"I don't want it to affect his brain, that's the thing...paralyzed I can handle." 

In keeping with the trend of variations in experience, not all patients viewed intellectual and cognitive deterioration as necessarily negative, with at least one caregiver reporting that a blunted affect would aid the patient to not experience the emotional consequences of dying.

"I find the hardest things now is for her to feel the emotional pain, whereas if she had no memory she wouldn't have the emotional worry."

### Attitudes towards euthanasia vary

Consistent with the contentious nature of this issue, our interviews did not point to a resounding consensus with regards to access to physician assisted suicide and euthanasia. However, although patients and caregivers were not united with respect to the means or the regulation of the practice, there was general agreement that the decision should ultimately be in the hands of the patient and family.

"...quality of life is important and I think when people think they've had enough, that that should be respected."

Most caregivers when asked directly supported the idea of euthanasia. They usually framed their responses in terms of quality of life and emphasized that withdrawal of care, for example, would be justified if the circumstances were grim. Some individuals, however, reported feeling uncomfortable with the idea of euthanasia, even in extreme cases. Others appealed to higher power.

"It may be right for some people and in some extreme cases...but for me personally I don't think I have the right, either for myself or for somebody else, to do that."

"That's something that God decides."

### Talking is helpful

Both patients and their loved ones were generally grateful for the opportunity to speak to a member of the health care team about these personal and distressing issues. Existential concerns are not typically broached in a clinical setting outside of palliative care wards, and caregivers especially commented that such studies will hopefully encourage health care workers to pay more individual attention to terminally ill patients.

" [It's important] for people to try to understand what people are going through. I think it's just as important as the medical treatment."

In more than one instance, an interview transcript could have been interpreted as a cathartic experience for a caregiver, who had been coping with a dying loved one and who viewed the opportunity to speak openly as almost therapeutic. Such interviews allowed them to reframe their experiences, and articulate to the medical world at large the importance of family centered care, and the uniqueness of the patient experience.

"When you're dealing with a terminal illness, it's not just about treating the patient but it's also about making things easier for the surviving family."

"I think it's extremely important. It helps people understand, if they have to go through it, to understand their feelings."

At the end of each interview, participants were asked what they thought of such studies and whether they perceived them as valuable. Only one patient, out of twenty nine participants did not see the potential utility of the study, but was able to understand and agree with it after it was explained to him in more practical terms. The overwhelming majority of patients viewed the interview as not only helpful but necessary, particularly in a health care climate that they perceived as more focused on the bottom line than helping individual patients.

"There is still someone who cares enough to ask about a family or a patient in a society where everything is governed by a budget."

"That's the thing about this study, is that somebody actually cares about my dad."

### Summary of themes

In order to facilitate the interpretation of the themes identified we created a coding framework that allowed us to further analyze the attitudes of respondents to each of the themes. When a caregiver or patient expressed strong feelings in favour of a given theme, such as that quality of life was extremely important, the value of +2 was assigned. When the feelings were milder, +1 was assigned and if feelings were completely neutral, a value of 0 was given. Similarly, when participants expressed strong feelings against a given theme, for example strongly believing that brain cancer was not unique, the value of -2 was assigned, and lighter negative feelings received -1.

**Figure 2:**
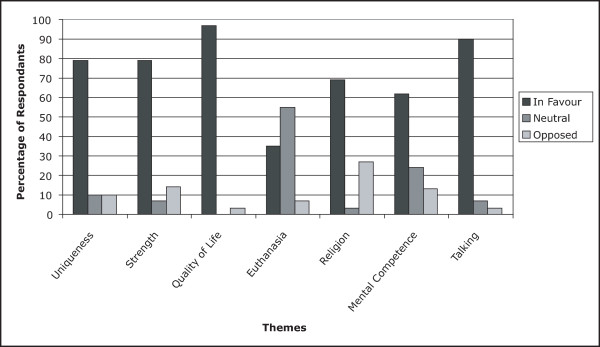
**Summary of Attitudes**. Graph illustrating the attitudes of patients and caregivers towards the seven identified themes. Attitudes were deemed to be in favour if they received a positive score (+2 or +1) according to our coding framework. Attitudes opposed were those that received negative scores (-2 or -1). A neutral score (0) was obtained if the participant was undecided, or if a question was not asked. Legend: *Uniqueness *– Brain Cancer is Unique; *Strength *– personal strength or lessons are derived from the illness experiences; *Quality of Life *– quality of life is more important than prolongation of life; *Euthanasia *– general attitude towards euthanasia; *Religion *– spirituality and/or religion play a role in coping with the illness; *Mental Competence *– a premium is placed on cognition, memory and mental competence over motor functioning; *Talking *– it is helpful to talk about the illness experience, and such studies are important.

Such a coding framework allowed us to understand the intensity with which participants viewed each theme, and highlighted in a novel way, the direction of the attitudes towards the illness experience. The results appear in Figure 2 and include the proportion of respondents who expressed favourable, opposing, or neutral thoughts towards each of the aforementioned themes. Several interesting points can be identified. Firstly, patients almost universally valued quality of life versus life prolongation when making treatment decisions. They also overwhelmingly perceived the interview experience to be not only beneficial but therapeutic, and saw such studies as potentially helpful for the development of comprehensive management of terminally ill patients. Respondents appeared to be relatively polarized with respect to the role of spirituality and religion, with few expressing neutral opinions, and nearly 70% finding solace in religious beliefs while 30% finding religion to not be helpful. Predictably, euthanasia proved to be the most contentious issue; however, relatively few expressed strong negative feelings towards it, with several participants justifying the action based on dwindling quality of life.

## Discussion

The aim of this study was to examine the values and attitudes of patients and their caregivers towards their experience with terminal brain cancer, and whether these influenced their treatment decisions. Our brains, and minds, differentiate us not only from other animals, but also from each other, and the opportunity to study the unique experiences of brain cancer patients is an opportunity that should not be missed.

A study examining the value of speaking to terminally ill patients about death and dying illustrated that such discussions are not only devoid of stress for participants but indeed are helpful [19]. In this study, approximately 50% of both patients and caregivers found discussions related to death and dying helpful and only less than 10% of patients, and less than 25% of caregivers reported the interview as stressful. Clearly such discussions, which promote communication both between patients and the health care team and among families are important, and should be taking place. Given the relative rapidity of the decline in many brain cancer patients, an argument can be made that the opportunities for discussion are fewer and hence, more urgent, in these patients, whose life has been drastically changed over so little time.

The novel aspects of the study here focused on the patient population and the application of qualitative analytic research methods. Recently, qualitative analysis was used in a palliative care setting to assess the attitudes of seven patients with terminal cancer who expressed a desire for hastened death [31]. The themes identified by that study echo some of our own, with patients focusing on the importance of a social support network and the derivation of life lessons and strength from their disease. The authors did not differentiate the patients according to cancer type, and so it us unknown whether any were suffering from brain cancer.

The themes we identified speak to the concerns and unique experiences of brain cancer patients. As with other malignancies, there are lessons to be learned from the unique aspects of organ specific diseases. For example, the association of breast resection with feelings of loss of femininity [32,33] may be viewed as analogous to the loss of cognitive functioning and identity seen with brain cancer. The fact that patients independently identify the differences between brain cancer and other malignancies reinforces the unique needs of patients suffering from an illness that affects their organ of self-identity and mind.

The finding that most patients and caregivers derived strength from their experiences is a testament to their resilience in the face of significant adversity. We believe that this resilience is more common than believed, and should be assumed when discussing diagnosis and treatment with patients and caregivers. However, as will be discussed below, there is an inherent selection bias in studies such as ours whereby emotionally stronger patients and caregivers may be more willing to discuss these difficult issues. This is a bias that is common to most research in palliative and chronic care [19,34].

An additional important pattern that emerged when patient and caregiver interviews were analyzed was that responses were frequently framed with respect to others, be they other caregivers, family members, or the patients' themselves. Interestingly, this pattern appeared to be common to all the themes identified; for example attitudes towards euthanasia and quality of life were frequently expressed in terms of what was best for others, rather than for the individual answering. This pattern underscores the complex interpersonal dynamic of the illness experiences, illustrating that decisions, and diagnoses, do not exist in vacuums, and that numerous people can be affected.

### Clinical relevance and recommendations

**Figure 3:**
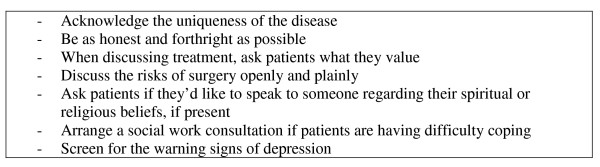
Recommendations for addressing illness experience issues with patients.

Our findings have direct clinical implications. Clinicians and health care providers, from neurosurgeons to ward nurses to social workers, all come into contact with patients with malignant disease, and eventually with patients with brain cancer. It is vitally important to understand the mindset of these patients and their caregivers in order to provide them with comprehensive and effective care. The themes identified here should provide a starting point from which fruitful discussions of the patient experience may begin, and on which treatment and management decisions can be premised. We have summarized our findings in the form of several recommendations in Figure 3. These recommendations serve to raise the consciousness of health care providers to these important issues.

Clinicians should first acknowledge the uniqueness of the disease. Patients and caregivers should have their concerns regarding personality change and cognitive decline validated, and it should be noted that these concerns are unique to brain cancer and that additional supports are available as needed. To this end, clinicians should arrange social work consultations for patients who they believe require additional assistance with dealing with the social consequences of their disease. In some cases a referral to psychology, or psychiatry, may be necessary.

Although religious and spiritual beliefs are common and varied, the clinician should not feel obligated to discuss these at great length. At the discretion of the individual physician, an acknowledgment of the consolation that religion can provide some patients may be helpful, and deepen the therapeutic alliance.

Perhaps most importantly, our study has demonstrated the importance of assessing patients' values and priorities with regards to treatment. Some patients place a premium on cognitive versus physical abilities, and will forego treatment that will spare motor activity but will risk memory or personality, for example. Other patients will choose the opposite. The nature of quality of life, therefore, is diverse and should be respected. The importance lies in asking patients what is important to them.

### Limitations of the study

Our study has several limitations. As alluded to earlier, there exists a selection bias, whereby only patients and loved ones who are strong enough emotionally and psychologically to discuss these personal issues will participate. Patients and caregivers who may not have come to terms with their diagnosis, or who are having a particularly difficult time dealing with their illness may be too distraught to participate. These patients are also arguably the most interesting, as the reasons for a failure to cope are sometimes more informative than an individual person's coping mechanism. Furthermore it would have been preferable to have more actual patients but this was challenging for obvious reasons.

Another weakness lies in our potentially limited representation of patient experiences. Although some studies have discussed the use of proxy substitutes to assess patient concerns and beliefs [35,36], there remains no real substitute for the patient's own words. Brain cancer, as we hoped to illustrate, is an intensely interpersonal disease that places both patients and caregivers in unique positions. One can argue, as we have, that the illness experience cannot be understood without examining both the patient and family perspectives, given the rapidity of the decline and the all-encompassing clinical nature of the disease. Nevertheless, we hope that future research will involve greater numbers of patients and will supplement the findings of the present study.

Further, since we designed the interview guideline, there exists the possibility that the best questions were not asked and that particular avenues were not explored, although the interview guide may (and did in this study) evolve over time.

The aim of this investigation, which was designed as a pilot study, was to describe our patients' and their loved ones' experiences with their illness rather than to arrive at broad, generalizable conclusions about those experiences. Our study naturally has several extensions that would enrich the results obtained here. Future studies can compare the themes discovered here to those obtained in other terminally ill patient populations, such as those dying of breast, or lung cancer. Furthermore, each of the themes obtained could suitably be used as a springboard for further investigation, for example, exploring in greater detail the psychological growth and lessons learned from coping with a terminal diagnosis. We hope that the issues and themes raised can stimulate further research in this important area.

## Conclusion

The importance of existential issues in terminally ill patients should not be underestimated. This importance, furthermore, is heightened in patients with advanced brain cancer, a disease that may affect the capacities that make patients unique, such as their personality and memory. We have identified several underlying themes in the illness experience of patients and caregivers that could serve as platforms on which discussions, such as those surrounding treatment, can be based. To address these issues and to keep them at the forefront of one's mind, would enrich the clinical experience and ensure that the patient and caregiver perspective is always respected. Arguably, our most important finding is that patients and their loved ones value the opportunity to engage in discussions about these delicate and sensitive issues as they approach the end of their life.

## Competing interests

The author(s) declare that they have no competing interests.

## Authors' contributions

NL helped design the study, performed data analysis and drafted the manuscript. AS conducted the interviews and performed subsequent analysis. JK assisted in refining the manuscript and conducted data analysis. MB conceived of the study, helped in its design, as well as edited and refined the manuscript

## Pre-publication history

The pre-publication history for this paper can be accessed here:


